# Adherence to antiretroviral therapy during pregnancy and the first year postpartum among HIV-positive women in Ukraine

**DOI:** 10.1186/1471-2458-14-993

**Published:** 2014-09-24

**Authors:** Heather Bailey, Claire Thorne, Ruslan Malyuta, Claire L Townsend, Igor Semenenko, Mario Cortina-Borja, the Ukraine European Collaborative Study Group in EuroCoord

**Affiliations:** Population Policy and Practice Programme, UCL Institute of Child Health, University College London, 30 Guilford Street, London, WC1N 1EH UK; Perinatal Prevention of AIDS Initiative, Odessa, Ukraine

**Keywords:** HIV, Antiretroviral therapy, Adherence, Self-efficacy, Ukraine, Eastern Europe, Women, Pregnancy, Prevention of mother-to-child transmission

## Abstract

**Background:**

Poor adherence to antiretroviral therapy (ART) is associated with HIV disease progression and, during pregnancy, increased mother-to-child transmission risk. In Ukraine, access to combination ART is expanding but data on adherence are scarce.

**Methods:**

Cross-sectional surveys of HIV-positive women were conducted i) at delivery (on antenatal ART adherence) and ii) during the first year postpartum (on ART adherence in the preceding four weeks). Factors associated with a score ≤11 on the self-report Case Adherence Support Evaluation (CASE) index or ≥1 self-reported missed dose were assessed using Fisher’s exact test.

**Results:**

Of 185 antenatal participants and 102 postnatal participants, median ages were 27.5 and 29.5 years respectively: 28% (50/180) and 27% (26/98) reported an unplanned pregnancy, and 13% (24/179) and 17% (17/98) an illicit drug-use history (excluding marijuana). One quarter (49/180 antenatally, 27/101 postnatally) screened positive for depression. The proportion reporting ‘low’ ART-related self-efficacy (i.e. unable to do ≥1/5 ART-taking activities) was 20% (28/141) antenatally and 17% (11/66) postnatally. Antenatally, 14% (95% CI 10-21%) had a CASE score ≤11 and 35% (95% CI 28-42%) reported missing ≥1 dose. Factors associated with a CASE score ≤11 were unplanned pregnancy (25% (12/48) vs. 11% (13/120) where planned, *p* = 0.03) and living with extended family (23% (13/57) vs. 10% (12/125) living with partner/alone, *p* = 0.04). Self-report of ≥1 missed dose antenatally was additionally associated with younger age (*p* = 0.03) and lower self-efficacy (50% (14/28) reported ≥1 missed dose vs. 28% (30/108) of those with high self-efficacy, *p* = 0.04). Of 102 postnatal participants, 8% (95% CI 4-15%) had a CASE score ≤11 and 31% (95% CI 22-41%) reported ≥1 missed dose. Of 11 women with low self-efficacy, 3 (27%) had a CASE score ≤11 compared with 3/55 (5%) of those with high self-efficacy (*p* = 0.05). Current smokers more commonly reported ≥1 missed dose postnatally (50% (13/26) vs. 25% (18/72) of non-smokers, *p* = 0.03).

**Conclusions:**

Our results highlight unmet needs for counselling and support. We identify some groups at risk of poor ART adherence, including women with markers of social vulnerability and those with low ART-related self-efficacy, who may benefit from targeted interventions.

## Background

Ukraine is a lower middle-income country which, along with Russia, accounts for around 90% of new HIV infections in Eastern Europe [1] – a region with an injecting drug use (IDU)-driven epidemic which continues to accelerate, in contrast with worldwide trends [2]. Newly diagnosed HIV infections in Ukraine are now equally distributed between women and men with adult HIV prevalence >1% overall [3]. In 2011, AIDS-related morbidity and mortality rates were 8.2 and 20.1 per 100,000 population respectively and 44% of AIDS diagnoses were in individuals not previously aware of their HIV infection [3].

Scale-up of antiretroviral therapy (ART) has been slow, but access to combination ART (cART) for HIV-positive pregnant women in Ukraine expanded following the shift in 2007 from a World Health Organisation (WHO) Option A policy (i.e. cART for women with treatment indications and zidovudine monotherapy (ZDVm) for those who require ART for prevention of MTCT (PMTCT) only) to an Option B policy (i.e. cART for all HIV-positive pregnant women) [4]. Antenatal cART coverage reached 60% in 2010 and 80% in 2011 [3, 5]. Access to cART among the general population is also improving: of ART-eligible individuals in HIV care in 2011, around 70% were receiving cART [3], up from 48% in 2009 [6]. The success of expanded cART coverage in improving health outcomes depends on adherence to treatment, low levels of which are associated with HIV disease progression, mortality and development of *de novo* resistance to antiretroviral drugs [7–9]. During pregnancy, a compromised virological response to cART also increases risk of mother-to-child transmission (MTCT) of HIV [10, 11], including of drug resistant virus [12]. Lower levels of adherence to ART during pregnancy have elsewhere been associated with use of illicit drugs, alcohol and tobacco [13–15], although not consistently [16, 17], possibly because of the importance of context-specific factors, particularly availability of harm reduction services [18]. Access to opioid substitution therapy in Ukraine is poor, with only 6,632 of an estimated 250,000 people injecting opiates receiving methadone or buprenorphine by the end of 2011 [3].

Information on adherence to ART during pregnancy and postnatally is essential for the strengthening of PMTCT and treatment programmes in Ukraine, where second-line ART options are currently lacking [19]. To our knowledge, there are no published data on ART adherence in Ukraine and little information available on adherence more widely in Eastern Europe, particularly among childbearing women, who may have different levels of and motivations for adherence than the general population [20]. In other settings, the immediate postpartum period has been found to be a time of high risk for poor ART adherence [13, 14, 21–23]. The aim of this study was to investigate levels and determinants of self-reported ART adherence during pregnancy and the first year postpartum among women with HIV in Ukraine.

## Methods

The European Collaborative Study (ECS) is an observational cohort study of HIV-infected pregnant women and their infants, active in Ukraine since 2000. At the time of this study, the ECS was enrolling around 30% of HIV-positive pregnant women in Ukraine at seven regional HIV/AIDS centres [5]. Linked anonymous data are collected on maternal and delivery characteristics and infant infection status, following informed consent.

For this investigation of ART adherence during pregnancy and postnatally, two cross-sectional surveys were conducted. The antenatal survey took place at three maternity hospitals among women who had taken ≥4 weeks of antenatal ART, who were invited to participate during their postnatal hospital stay (typically of ≥3 days). The postnatal survey took place at six HIV/AIDS centres among women attending between 1 and 12 months postpartum for routine infant follow-up and ongoing HIV care, who were currently on ART. See the Acknowledgements section for a full list of participating sites and information on ethics and institutional approvals. Survey participation was open to all women meeting the eligibility criteria, regardless of whether they were enrolled in the ECS. Participants gave informed consent and completed a paper-based questionnaire. Unique study identifiers allowed clinicians to refer women for additional support where necessary, while ensuring participant anonymity to researchers.

The survey period was from July to December 2011, with extension to April 2012 for sites with operational problems (e.g. periods of ward closure). We constructed indirect measures of survey participation for the main six-month study period. For the antenatal survey, the number of surveys returned was calculated as a proportion of the 240–300 deliveries to HIV-positive women at participating hospitals over the main six-month survey period (of whom 90% were estimated to be eligible [5]). For the postnatal survey, data from a postnatal cohort nested within the ECS on number of women attending HIV care postnatally were used to calculate participation rates. There were fewer postnatal than antenatal participants because only around a fifth of women continue ART after delivery in Ukraine, with many of the remainder not yet meeting eligibility criteria for treatment [24]. Survey responses were matched to ECS data using maternal date of birth, date of delivery, infant sex and centre, and characteristics of survey participants compared with those of women who did not participate to explore the survey population’s representativeness. Associations between adherence and clinical characteristics were explored in the subgroup with ECS data available. Data collected in the adherence surveys included ART adherence, socio-demographic characteristics, health behaviours, disclosure of HIV status, experience of ART side effects, reasons for missed doses of ART (from the AIDS Clinical Trials Group adherence tool [25]), symptoms of depression and ART-related self-efficacy. Women’s perceptions of the risks and benefits of ART for PMTCT were explored using questions from the NIAID Adult AIDS Clinical Trials Group supplemental antepartum adherence questionnaire [26]. Postnatally, women were asked if and how their adherence had changed following delivery (question adapted from Mellins *et al.* [13]).

### Definitions

Self-reported adherence to ART was measured using the Case Adherence Support Evaluation (CASE) index tool. Answers to three questions (on difficulty taking medications on time, frequency of missed doses, and timing of last missed dose) give a score of between 3 and 16, with higher scores indicating better adherence [27]. Previous studies validating this tool against viral load have determined best score cut-offs for defining ‘poor’ ART adherence to be ≤10 [27], ≤11 [28] and ≤12 [29]; in this study, a score of ≤11 was chosen as it showed the greatest association with viral load measures in validation analyses (data not shown). Responses were also categorised according to whether any missed doses were reported (one potentially valid approach [30]); ‘≥1 missed dose’ referred to the whole of pregnancy in the antenatal survey, and the preceding four weeks in the postnatal survey.

Symptoms of depression over the last month were measured using Patient Health Questionnaire-2 Screening Questions from the Primary Care Evaluation of Mental Disorders [31], with a positive depression screening test result defined as self-reported anhedonia and low mood, or one of these and a positive response to an enquiry about the need for help [32].

ART-related self-efficacy was measured using five questions adapted from the HIV Treatment Adherence Self-Efficacy Scale [33]. Participants indicated their level of confidence from ‘could not do’ [0 points], ‘fairly confident I could do’ [1 point] and ‘confident I could do’ [2 points]. Women answering all five questions and scoring <5 were defined as having low self-efficacy. Two questions about help-seeking self-efficacy were also included.

### Data analysis

Univariable comparisons were assessed with the χ^2^ test and the Fisher’s exact test for categorical variables and the Wilcoxon Mann–Whitney test for medians; unless otherwise stated, *p*-values were obtained using two-sided Fisher’s exact tests. Multivariable analyses were not conducted, because the number of participants and inter-correlation of many factors associated with poor adherence meant that statistical power was insufficient to adjust for confounding. The aim of this work was to characterise women likely to benefit most from targeted adherence support, rather than to establish the relative importance of independent factors. Data were managed using REDCap electronic data capture tools hosted at University College London Institute of Child Health [34]. Statistical analyses were performed using STATA version 11.0 (StataCorp, LP, College Station, Texas, USA).

## Results

A total of 287 women participated in the surveys (185 in the antenatal and 102 in the postnatal survey); seven women completed both surveys. Participant characteristics are shown in Table 1. Of the 185 antenatal study participants, 106 took part during the main six-month survey period, giving a participation rate among eligible women of 39-49% (based on 240–300 HIV-positive women delivering at participating hospitals in this period, of whom an estimated 90% - i.e. 216–270 - had taken ≥4 weeks of antenatal ART and thus met eligibility criteria). Of the total 102 postnatal participants, 63 took part during the main six-month survey period at five HIV/AIDS centres with denominator data available; these 63 women were estimated to represent 75% (63/84) of women on ART at their first visit to the HIV/AIDS centre following delivery.

**Table 1:** **Characteristics of survey participants**

	Antenatal survey (***n*** = 185)	Postnatal survey (***n*** = 102)
**Median age at participation (IQR)**	27.5 years (25.0, 30.9)	29.5 years (25.9, 33.5)
**Median time since delivery (IQR)**	1 day (1, 2)	5.3 months (2.4, 7.8)
**Marital status**		
Married	100 (54%)	55 (54%)
Cohabiting	51 (28%)	23 (23%)
Single	34 (18%)	24 (24%)
**Pregnancy unplanned**	50 (28%)	26 (27%)
**Living with** ^**†**^		
Husband/partner	132 (72%)	80 (79%)
Extended family (including parents)	60 (33%)	23 (23%)
Living as only adult in household	12 (7%)	13 (13%)
**Disclosure of HIV status** ^**†**^		
To husband/partner	135 (74%)	80 (79%)
To family or friend(s)	108 (59%)	55 (54%)
To no one at all	16 (9%)	7 (7%)
**Do you have someone you can rely on to help you care for your baby?**		
Yes	164 (91%)	89 (88%)
No	17 (9%)	12 (12%)
**Smoking, alcohol and drug use** ^**†**^		
Current smoker	49 (27%)	28 (28%)
Current alcohol use	168 (9%)	8 (8%)
Ever used marijuana	25 (14%)	15 (15%)
Ever used illicit drugs other than marijuana	24 (13%)	17 (17%)
**Severity of ART side effects**		
Not bothered by side effects or only slightly	132 (75%)	76 (76%)
Somewhat or terribly bothered by side effects	44 (25%)	24 (24%)
**Use of support services** ^**†**^		
Currently using support group	10 (5%)	21 (21%)
Currently using peer counselling	10 (5%)	27 (26%)
Currently using social services	40 (22%)	28 (27%)
Currently using adherence programme	4 (2%)	39 (38%)

^**†**^Groups not mutually exclusive.

Postnatal participants completed the survey a median of 5.3 months after delivery, and were a median of two years older than antenatal participants. Around three-quarters in each group were living with a husband or partner (Table 1). In the antenatal survey, 15% (*n* = 20) of the 132 women living with a husband or partner were also living with other adult family members, while in the postnatal survey this proportion was 14% (11/80). Overall, women living with their family were younger (median age 26.6 years vs. 28.6 years for women living only with a partner or alone in the antenatal survey, Wilcoxon-Mann–Whitney test *p* = 0.02) and more likely to have had an unplanned pregnancy (36% (21/58) vs. 23% (27/120) of those living only with a partner or alone, χ^2^ = 3.73 *p* = 0.05). This was also the case in the postnatal survey group, although comparisons were not statistically significant; women living with their family had a median age of 27.9 years vs 29.9 years for the rest of the group (Wilcoxon-Mann–Whitney *p* = 0.27), and 36% (8/22) had an unplanned pregnancy vs. 24% (18/75) of those living with a partner only or alone (χ^2^ = 1.33 *p* = 0.25).

**Table 2:** **Clinical characteristics and ART use among adherence study participants with matched ECS data available, and their representativeness with respect to women enrolled in the ECS**

	Adherence study		
	Antenatal survey and ECS (***n***=97)	Postnatal survey and ECS (***n***=44)	ECS enrolment only (***n*** = 1596)
**Age at leaving full-time education**			
≤16 years	1 (6%)	2 (9%)	204 (26%)
17 years or above	15 (94%)	20 (91%)	595 (74%)
**History of injecting drug use**			
No	87 (90%)	35 (80%)	1398 (88%)
Yes	10 (10%)	9 (20%)	191 (12%)
**History of pregnancy termination**			
0	40 (45%)	14 (39%)	540 (41%)
1	27 (31%)	8 (22%)	338 (26%)
2 or more	21 (24%)	14 (39%)	435 (33%)
**Timing of HIV diagnosis**			
Before pregnancy	44 (46%)	23 (52%)	693 (44%)
1st/2nd trimesters	48 (50%)	21 (48%)	736 (46%)
3rd trimester/intrapartum	4 (4%)	0	159 (10%)
**Antenatal CD4 count (first in pregnancy)**			
≤350 cells/mm^3^	19 (28%)	26 (76%)	484 (39%)
>350 cells/mm^3^	48 (72%)	8 (24%)	773 (61%)
**WHO stage**			
1-2	67 (78%)	25 (61%)	1160 (81%)
3-4	19 (22%)	16 (39%)	269 (19%)
**Timing of ART initiation**			
Before conception	7 (8%)	7 (17%)	95 (7%)
1st/2nd trimester	61 (66%)	23 (56%)	929 (65%)
3rd trimester	25 (27%)	11 (27%)	402 (28%)
**Antenatal ART regimen**			
Mono/dual	11 (12%)	3 (7%)	314 (22%)
cART	83 (88%)	39 (93%)	1139 (78%)
**Median weeks on ART by completion of adherence survey**	13.7 (IQR 11.3, 17)	43.7 (IQR 26.0, 54.9)	

ECS data were available for 97 (52%) of antenatal and 44 (43%) of postnatal participants at the time of analysis; clinical and treatment data on this sub-group are shown in Table 2, along with ECS data on women who did not participate in the adherence surveys. In the antenatal survey, 12% of women were taking mono/dual therapy (Table 2). Of those on cART and reporting their regimen in full, 91% (86/94) in the antenatal survey and 80% (47/59) postnatally were taking a ritonavir-boosted lopinavir-based regimen. Most women (antenatal: 72% (102/142), postnatal: 56% (40/72)) were taking six antiretroviral pills a day. Postnatal participants were more likely to have WHO stage 3–4 disease (39% (16/41) vs. 22% (19/86) of antenatal participants, χ^2^ = 3.99 *p* = 0.05) and had been taking ART for longer (Table 2, Wilcoxon Mann–Whitney test *p* < 0.01), reflecting their indications for treatment rather than for PMTCT only. Of note, adherence study participants had higher levels of education compared with women enrolled in the ECS only (92% (35/38) remained in full-time education until ≥17 years of age vs. 74% (595/799) of women in the ECS only, Fisher’s exact test *p* = 0.01).

A quarter of women screened positive for depression (49/180 antenatally and 27/101 postnatally). With regards ART-related self-efficacy, the area in which women most commonly lacked confidence was in their ability to keep taking medication if experiencing side effects (Table 3). Most reported being able to share concerns with their clinician, but fewer were able to ask someone for support with taking their medication (Table 3). Although most antenatal survey participants were completely (44%, 80/180) or fairly (51%, 91/180) sure that antenatal ART was effective for PMTCT, 82% (149/181) were at least a little worried that it might harm their baby. Women who were completely sure of the effectiveness of ART were less likely to be worried about harm (74% (59/80) vs. 88% (80/91) of those who were only fairly sure of its effectiveness, χ^2^ = 5.61 *p* = 0.02).

**Table 3:** **Measures of self-efficacy**

	Antenatal survey			Postnatal survey		
	Confident could do	Fairly confident could do	Could not do	Confident could do	Fairly confident could do	Could not do
**ART-related self-efficacy**						
1. Keep taking ART if side effects interfere	40% (68/171)	46% (78/171)	15% (25/171)	29% (26/89)	51% (45/89)	20% (18/89)
2. Keep taking ART in front of people unaware of your HIV status	43% (75/175)	46% (81/175)	11% (19/175)	31% (27/88)	59% (52/88)	10% (9/88)
3. Keep taking ART if daily routine disrupted	45% (78/173)	47% (81/173)	8% (14/173)	41% (35/86)	57% (49/86)	2% (2/86)
4. Keep taking ART if not feeling well	43% (77/178)	47% (83/178)	10% (18/178)	24% (20/85)	62% (53/85)	14% (12/85)
5. Keep attending appointments if they interfere with daily activities	36% (54/148)	55% (82/148)	8% (12/148)	45% (32/71)	52% (37/71)	3% (2/71)
Proportion reporting that they could not do ≥1 of 5 ART-related items	20% (28/141)			17% (11/66)		
**Help-seeking self-efficacy**						
1. Ask clinician for more information or tell them about concerns or worries	37% (61/167)	56% (94/167)	7% (12/167)	48% (41/86)	49% (42/86)	3% (3/86)
2. Ask someone for support with taking medication if needed	33% (54/165)	47% (78/165)	20% (33/165)	30% (26/86)	43% (37/86)	27% (23/86)

### Prevalence of poor adherence and reasons for missed doses

Median CASE scores were 15 (of a possible 16) in both the antenatal survey (*n* = 173) and the postnatal survey (*n* = 99) (antenatal: IQR 13, 16, range 6, 16; postnatal: IQR 14, 16, range 7, 16). The proportion with a CASE score ≤11 (defined as ‘poor’ adherence) was 14% (25/173, 95% CI 10-21%) in the antenatal survey and 8% (8/99, 95% CI 4-15%) in the postnatal survey, while 35% (61/176, 95% CI 28-42%) and 31% (31/100, 95% CI 22-41%) reported having missed ≥1 dose during pregnancy and in the last four weeks respectively. Antenatally, the most common reasons for missing a dose were being away from home (8%, 14/185), forgetting (5%, 10/185) and feeling sick or ill (5%, 10/185), while postnatally these were forgetting (15%, 15/102), sleeping through the dose (9%, 9/102) and being away from home (8%, 8/102). In the postnatal survey, 13% (12/93) reported having missed fewer doses during pregnancy than postnatally, 2% (2/93) having missed fewer doses postnatally than when pregnant, 6% (6/93) having unchanged adherence, and 78% (73/93) having missed no doses in either time period. Of the 12 women who reported adhering to ART better during pregnancy than postnatally, 11 cited concerns about MTCT as a reason for this.

### Factors associated with poor adherence during pregnancy

Table 4 shows factors associated with a CASE score ≤11 and with reporting ≥1 missed dose during pregnancy. Women living with their extended family (a factor correlated with youth and unplanned pregnancy) were more likely to report poor adherence, particularly if they had not disclosed their HIV status to a family member (40% (8/20) of whom had a CASE score ≤11 vs. 14% (5/37) living with family who had disclosed their status to a family member, *p* = 0.04). Overall however, disclosure of HIV status to at least one other person (reported by 91% (167/183) of women) was not associated with adherence (Table 4). Among married/cohabiting women, 83% (124/150) had disclosed their HIV status to their partner, of whom 32% (37/116) reported missing ≥1 dose during pregnancy compared with 42% (11/26) who had not disclosed their HIV status to their partner (*p* = 0.36).

**Table 4:** **Factors associated with poor ART adherence during pregnancy**

	CASE score ≤11 points (25/173)	Fisher’s exact test	≥1 missed dose (61/176)	Fisher’s exact test
**Age**				
<25 years	26% (11/42)	*p* = 0.13	52% (23/44)	***p*** **= 0.03**
25-27 years	10% (5/48)		23% (11/48)	
28-30 years	12% (5/41)		29% (12/41)	
≥31 years	10% (4/42)		35% (15/43)	
**Marital status**				
Married	14% (13/93)	*p* = 0.96	34% (32/95)	*p* = 0.83
Cohabiting	15% (7/48)		33% (16/48)	
Single	16% (5/32)		39% (13/33)	
**Pregnancy planned**				
Yes	11% (13/120)	***p*** **= 0.03**	30% (36/122)	***p*** **= 0.02**
No	25% (12/48)		49% (24/49)	
**Lives with family**				
No	10% (12/115)	***p*** **= 0.04**	29% (34/117)	***p*** **= 0.04**
Yes	23% (13/57)		46% (26/57)	
**Lives with partner**				
No	18% (9/49)	*p* = 0.47	45% (22/49)	*p* = 0.08
Yes	13% (16/123)		30% (38/125)	
**Disclosed HIV status to anyone**				
No	20% (3/15)	*p* = 0.46	38% (6/16)	*p* = 0.79
Yes	14% (22/157)		35% (55/159)	
**Current smoker**				
No	14% (18/127)	*p* = 1.00	30% (39/129)	*p* = 0.07
Yes	14% (6/44)		47% (21/45)	
**History of drug use (other than marijuana)**				
No	14% (21/146)	*p* = 1.00	35% (52/149)	*p* = 1.00
Yes	14% (3/21)		33% (7/21)	
**Self-efficacy score**				
≥5 (higher self-efficacy)	12% (13/107)	*p* = 0.22	28% (30/108)	***p*** **= 0.04**
<5 (lower self-efficacy)	22% (6/27)		50% (14/28)	
**Positive depression screen**				
No	13% (16/124)	*p* = 0.33	31% (39/125)	*p* = 0.08
Yes	20% (9/46)		46% (22/48)	
**Can you ask doctor for support?**				
Certain/fairly certain	14% (21/155)	*p* = 0.06	33% (52/156)	*p* = 0.34
Cannot do	36% (4/11)		50% (6/12)	
**Antenatal ART**				
Zidovudine monotherapy	29% (4/14)	*p* = 0.23	64% (9/14)	***p*** **= 0.02**
cART	13% (19/141)		31% (44/143)	
**Severity of ART side effects**				
Not bothered or only slightly	14% (18/126)	*p* = 0.81	33% (42/127)	*p* = 0.71
Somewhat or terribly bothered	16% (7/43)		37% (16/43)	
**Knowledge of whether antenatal ART is effective for PMTCT**				
Completely or fairly sure that it is effective	12% (20/162)	*p* = 0.10	33% (54/165)	*p* = 0.17
Unsure that it is effective	33% (3/9)		56% (5/9)	

p values ≤0.5 are indicated in bold.

There was some evidence of an association between current smoking and reporting of ≥1 missed dose during pregnancy (*p* = 0.07), but not between history of illicit drug use and adherence (Table 4). A positive depression screening test result was weakly associated with increased likelihood of reporting ≥1 missed dose (Table 4). Adherence did not differ by whether ART was perceived as safe (*p* = 1.00 for ≥1 missed dose), but there was some evidence that adherence was poorer among the small group of women unsure of the effectiveness of ART for PMTCT (Table 4). Women who received ZDVm were more likely to report ≥1 missed dose compared with those on cART (Table 4, *p* = 0.02), while those who were confident to ask their doctor for support had higher CASE scores than those who reported that they could not do this (Table 4). However, there was no difference in adherence by severity of HIV disease (13% (8/64) and 11% (2/17) of women with WHO stage 1–2 and 3–4 disease had a CASE score of ≤11 respectively, *p* = 1.00; subgroup with ECS data).

### Factors associated with poor adherence in the year following delivery

Factors associated with a CASE score ≤11 or with reporting ≥1 missed dose in the last four weeks in the postnatal survey are shown in Table 5. Current smokers were more likely to report ≥1 missed dose, as were women with a history of illicit drug use and those not using a treatment adherence programme (*p* = 0.08 for latter two factors, Table 5). Women who had not disclosed their HIV status to anyone were more likely to report having missed ≥1 dose, and there was some indication of lower ART adherence among unmarried and single women (vs. married women) and those not living with a partner (Table 5), although small numbers limited statistical power. Women with low self-efficacy were more likely to have a CASE score ≤11, but there was no association between adherence and depression screening test result postnatally and severity of self-reported ART side effects was also not associated with adherence (Table 5). Of the subgroup with ECS data available, 36% (9/25) of women with WHO stage 1–2 disease reported having missed a dose in the last four weeks vs. 13% (2/16) of those with WHO stage 3–4 disease (*p* = 0.15).

**Table 5:** **Factors associated with poor ART adherence in the year following delivery**

	CASE score ≤11 points (8/99)	Fisher’s exact test	≥1 missed dose (31/100)	Fisher’s exact test
**Age**				
<25 years	9% (2/22)	*p* = 0.95	23% (5/22)	*p* = 0.37
25-27 years	7% (1/15)		19% (3/16)	
28-30 years	5% (1/21)		29% (6/21)	
≥31 years	10% (4/40)		40% (16/40)	
**Marital status**				
Married	6% (3/54)	*p* = 0.13	22% (12/55)	*p* = 0.08
Cohabiting	19% (4/21)		38% (8/21)	
Single	4% (1/24)		46% (11/24)	
**Lives with family**				
No	8% (6/75)	*p* = 1.00	30% (23/76)	*p* = 0.80
Yes	9% (2/23)		35% (8/23)	
**Lives with partner**				
No	10% (2/21)	*p* = 0.68	48% (10/21)	*p* = 0.11
Yes	8% (6/77)		27% (21/78)	
**Disclosed HIV status to anyone**				
No	14% (1/7)	*p* = 0.46	71% (5/7)	***p*** **= 0.03**
Yes	8% (7/91)		28% (26/92)	
**Current smoker**				
No	6% (4/71)	*p* = 0.20	25% (18/72)	***p*** **= 0.03**
Yes	15% (4/26)		50% (13/26)	
**History of drug use (other than marijuana)**				
No	8% (6/78)	*p* = 0.63	28% (22/79)	*p* = 0.08
Yes	12% (2/17)		53% (9/17)	
**Using treatment adherence programme**				
No	7% (4/60)	*p* = 0.71	38% (23/61)	*p* = 0.08
Yes	10% (4/39)		21% (8/39)	
**Self-efficacy score**				
≥5 (higher self-efficacy)	5% (3/55)	***p*** **= 0.05**	36% (20/55)	*p* = 0.32
<5 (lower self-efficacy)	27% (3/11)		55% (6/11)	
**Positive depression screen**				
No	7% (5/71)	*p* = 0.68	28% (20/72)	*p* = 0.46
Yes	11% (3/27)		37% (10/27)	
**Severity of ART side effects**				
Not bothered or only slightly	8% (6/74)	*p* = 1.00	29% (22/75)	*p* = 0.46
Somewhat or terribly bothered	8% (2/24)		38% (9/24)	

p values ≤0.5 are indicated in bold.

## Discussion

In this cross-sectional study of ART adherence in childbearing women in Ukraine, poor adherence (defined as a score of ≤11 on the CASE adherence index measure) was reported by 14% (95% CI 10-21%) of women during pregnancy and 8% (95% CI 4-15%) of women in their first year postpartum. A third of women in the antenatal survey reported missing at least one dose during pregnancy, and a similar proportion postnatally reported missing at least one dose in the preceding four weeks. Cross-study comparisons of ART adherence are problematic due to the range of assessment methods and outcomes used; a recent meta-analysis estimated that 72% of women took >80% of ART doses during pregnancy [20], but self-reported measures (as used in this study) are generally inflated compared with more objective measures [35–37]. Despite fairly high levels of adherence in our study, most women (>80%) were worried about the safety of antenatal ART, a quarter screened positive for depression, and one in four reported being somewhat or terribly bothered by ART side effects. Unmet need for support may impact on the sustainability of ART adherence beyond pregnancy and the first year following delivery.

Levels of adherence reported during and after pregnancy were similar, but there were important differences between the survey groups, particularly with respect to HIV disease stage, reflecting the fact that a large proportion of HIV-positive women stop ART after delivery in this setting, as they lack indications for treatment. Risk of disengagement from HIV care is high in the postnatal period, and women with risk factors for poor adherence may have been under-represented in our postnatal survey if they were also more likely to disengage from care after delivery. We therefore could not make meaningful comparisons of adherence between the two time periods. Women receiving antenatal ZDVm reported poorer adherence overall than those receiving cART, possibly because ZDVm is used preferentially among women with indicators of social disadvantage [5], and because women initiating cART may receive more intensive adherence counselling. ZDVm (WHO Option A) is no longer recommended by the WHO for PMTCT [38]; the shift in international guidelines towards lifelong cART for all pregnant HIV-positive women (WHO Option B+) [38] underscores the importance of adherence support continuing postpartum for the success of future treatment programmes [39]. Of note, a minority (12/93) of postnatal respondents reported a decline in adherence following delivery but only two reported an improvement. We were therefore not able to rule out poorer levels of adherence postpartum, as has been reported elsewhere [13, 14, 21–23].

Poor adherence during pregnancy was more commonly reported among women living with their extended family, women not living with a partner, younger women and those with an unplanned pregnancy (factors which were inter-related). Lack of financial support from a partner has been associated with poor ART adherence among women in South Africa [40], and younger age with increased risk of poor ART adherence and/or poor virological outcomes in the UK and North America [41–43], including among pregnant and postpartum women [16, 17]. Our findings on the association between poor adherence and lack of disclosure to family members among the sub-group of women living with their extended family highlight the importance of providing support for disclosure within the household. Of note, 1 in 10 women reported being unable to take ART in front of someone who was unaware of their HIV status.

Women with other specific poor health behaviours were more likely to report poor adherence. This included current smokers, who were more likely to report having missed a dose (statistically significant only postnatally), and are a generally less affluent group [44]. There was some evidence of an association between history of drug use and poor ART adherence postnatally but not during pregnancy in our study. IDUs engaged with PMTCT interventions (and particularly those having taken antenatal ART for ≥4 weeks – an inclusion criteria for this study) may be a select group better able to adhere than those receiving ART for their own health postnatally.

Lower ART-related self-efficacy was associated with self-report of ≥1 missed dose during pregnancy and a CASE score ≤11 postnatally. Women with a positive depression screening test were also slightly less likely to report complete antenatal adherence (*p* = 0.08). Cognitive-behavioural interventions to increase self-efficacy have been associated with reduced depressive symptoms, increases in CD4 count and decreases in viral load among HIV-positive women in the US [45, 46], and are a potential area for intervention in this population. CASE scores ≤11 were more common antenatally among women not confident to seek information and support from their doctor. The time needed to build a positive, trusting relationship with a healthcare provider, of potential importance to adherence behaviours [47, 48], is limited before initiating ART during pregnancy. Pregnant women have specific counselling needs regarding ART preparedness – e.g. the exacerbation of side effects by physical changes during pregnancy [14] – and our results highlight areas for improvement, particularly around the risks and benefits of ART. As the number of HIV-positive pregnant women in Ukraine increases and ART coverage expands [3, 5], strategies are needed to ensure sustainable delivery of high-quality counselling. Support services were most commonly used in our sample after delivery, suggesting that access could be improved in pregnancy. Women using treatment adherence programmes were more likely to report good adherence postnatally, but further work is needed to assess the impact of these programmes in specific groups.

This study is limited by its use of a self-report adherence measure which, while shown to be associated with virological response [27–29], is likely to over-estimate actual levels of adherence. The sample size of the surveys and the lack of variability in CASE scores limited statistical power to detect differences in ART adherence by maternal characteristics, particularly postpartum, as the proportion of women eligible to continue ART after delivery and their long follow-up intervals and loss to follow-up [24] restricted the number of women eligible and available to participate. The trends in characteristics of participants who reported ≥1 missed dose (the more sensitive of the two outcome measures studied) were broadly similar to those with a CASE score ≤11 points in the antenatal survey, with the exception of smokers and those not living with a partner, for whom an increased risk of ≥1 missed dose was detected but not an increased risk of having a CASE score ≤11 points. In the postnatal survey, women not living with a partner were also at increased risk of ≥1 missed dose but not a CASE score ≤11 points, as were women who had not disclosed their HIV status to anyone, single women, those with a history of drug use, and those not using a treatment adherence programme. This partly reflects greater statistical power to detect differences by reporting of ≥1 missed dose compared with CASE score ≤11 (due to the larger number of women reaching the more sensitive cut-off), but possibly also the characteristics of women with early indications of adherence problems.

Participation rates were estimated at 39-49% of eligible women in the antenatal survey and around 75% of eligible women on ART in the postnatal survey. Importantly, some women most at risk of poor adherence may have been excluded due to our eligibility criteria (of note, survey participants were more highly educated than women enrolling only in the ECS) and we may therefore have over-estimated adherence levels in the populations of interest.

Our study design and the use of both WHO Option A and Option B strategies for women requiring ART for PMTCT precluded comparison of adherence during pregnancy and after delivery; future longitudinal studies are needed to assess changes in adherence over time in the subgroup of women who have treatment indications for their own health and continue ART postnatally.

## Conclusions

Our results highlight unmet needs for counselling and support and identify some risk groups – particularly women with markers of social vulnerability, substance users and those with low ART-related self-efficacy – who may benefit from targeted interventions to support ART adherence during pregnancy and postnatally.

## Abbreviations

ART: Antiretroviral therapy
cART: Combination antiretroviral therapy
CASE: Case adherence support evaluation
ECS: European Collaborative Study
MTCT: Mother-to-child transmission
PMTCT: Prevention of mother-to-child transmission
WHO: World Health Organisation
ZDVm: Zidovudine monotherapy.
